# A review of childhood rotavirus vaccination policies and a presentation of vaccine coverage trends at national and regional level, Italy, 2016 to 2023

**DOI:** 10.2807/1560-7917.ES.2025.30.39.2500026

**Published:** 2025-10-02

**Authors:** Sara Farina, Alessandra Maio, Andrea Zaino, Luigi Russo, Walter Ricciardi, Roberto Ieraci, Leonardo Villani

**Affiliations:** 1 Section of Hygiene, University Department of Life Sciences and Public Health, Università Cattolica del Sacro Cuore, Rome, Italy; 2 Regione Lazio – Strategie Vaccinali, Rome, Italy; 3 UniCamillus - Saint Camillus International University of Health and Medical Sciences, Rome, Italy; * These authors contributed equally to this work and share first authorship.

**Keywords:** s: rotavirus, vaccination coverage, Italy, immunization, health policy

## Abstract

**BACKGROUND:**

Rotavirus is a main gastroenteritis cause in children ≤ 5 years old. In 2017, when few Italian regions had rotavirus vaccination programmes, rotavirus vaccines were included in the National Plan for Preventive Vaccination (PNPV). Although all Italian regions follow the PNPV, they each decide how to implement it, contributing to rotavirus vaccination coverage differences across the country.

**AIM:**

The objective was to assess rotavirus vaccination national/regional policies in Italy and, between 2016 and 2023, vaccination coverage trends at national and regional level.

**METHODS:**

Scientific and grey literature was systematically reviewed for reports on Italian national/regional policies or programmes concerning rotavirus vaccination. Their key features and strategies to increase vaccination coverage were recorded. Vaccination coverage data originating from the Ministry of Health, were analysed descriptively, or with linear regression, for national and regional trends.

**RESULTS:**

Among 418 policy/programme reports identified, 25 were included. Between 2013 and 2015, Sicilia, Calabria and Puglia had already initiated universal vaccination programmes. The PNPV 2017–19 standardised regions’ offer of rotavirus vaccination. Between 2016 and 2023, vaccination coverage in Italy significantly increased (p = 0.0005) from 10.5% to 70.76%, with a 140% rise in 2019−20. Regional coverage disparities existed. Throughout 2016–23, most central Italian regions had annual coverages below national values. Bolzano annual coverage was consistently < 50%, while in Veneto, coverage reached 85.10% in 2021. In 2023, five regions had > 80% coverage.

**CONCLUSIONS:**

While rotavirus vaccination coverage improved in Italy in 2016−23, regional disparities persist. Addressing these requires overcoming logistical and societal challenges, as well as harmonised policies.

## Introduction

Rotavirus (RV) is transmitted between people through the faecal–oral route, either directly from person to person, or indirectly through contaminated surfaces and objects. Individuals who get infected by the virus, and who subsequently develop illness, typically experience a sudden onset of fever and vomiting, followed by profuse watery diarrhoea, often resulting in dehydration. Because of a risk that dehydration becomes life-threatening [1], especially for children aged 6–23 months [2], cases of severe disease may require hospitalisation.

Infections with RV contribute to high rates of morbidity and mortality in the world, representing a major public health challenge. In children less than 5 years old, RV infections are a principal cause of acute gastroenteritis [3], resulting in over 258 million episodes of diarrhoea globally in 2016 [4]. Moreover, RV enteritis is a leading factor of diarrhoeal death in this age group, where it accounted in 2015 for 146,500 such deaths worldwide (29.3% of total diarrhoeal deaths in that year) [4,5].

Low and middle-income countries are notably impacted by RV, particularly countries in the Sub-Saharan African region [6]. In this region, an estimate of 117 million RV infections in children under 5 years old in 2016 has been reported, as well as 104,733 related deaths [6]. While high-income countries are less vulnerable to RV infections, they are nonetheless also affected [7]. The European Centre for Disease Prevention and Control (ECDC) published in 2017 that based on studies conducted in 18 European Union/European Economic Area (EU/EEA) countries, there were annually between 300 and 600 children aged under 5 years per 100,000 hospitalised due to RV gastroenteritis in these countries [8]. By extrapolating these results to the whole EU/EEA, it was considered that 75,000 to 150,000 hospitalisations in under 5-year-olds were caused by RV on a yearly basis in the EU/EEA [8]. Another study released in 2006 found that among children younger than 5 years in the EU, RV was responsible for 3.6 million episodes of disease and nearly 700,000 outpatient visits annually [9]. Even though these numbers have been recently decreasing [10], the World Health Organization (WHO) Regional Office for Europe reported that between 2008 and 2016, 26,831 of ca 100,000 hospital admissions for acute gastroenteritis in 0–59-month-olds (ca 27.8%), stemmed from RV infection [11].

In Italy, the REVEAL study, conducted in 2004–05, showed that among children under 5 years old diagnosed with acute gastroenteritis, RV was responsible for 68.9% admitted to hospital, 61.3% admitted to the emergency department, and 32.9% seen in primary care settings [12,13]. Furthermore, despite an apparent decrease in the incidence of hospital discharges due to acute gastroenteritis in the country (from 16.6 per 100,000 inhabitants in 2009–13 to 9.9 per 100,000 inhabitants in 2018–19 [14]), a total of 79,344 hospitalisations for acute gastroenteritis were associated to RV in 2005–12 [13]. Adding to a negative public health impact, RV infections have a considerable economic effect, with direct costs for hospitalisations and medical visits, as well as indirect costs related to parental absence from work [15].

Vaccines for RV, which have been available since 2006 [16,17], have demonstrated a high efficacy in preventing gastroenteritis and have led to an important reduction in hospitalisations and emergency department visits in vaccinated populations [7]. In Italy, the National Plan for Preventive Vaccination (PNPV) serves as the national guideline for vaccination policy. A law adopted in 2017 [18], which increased the number of mandatory vaccinations from four to 10, also recommended other vaccinations including RV vaccination [19].

Two live-attenuated oral RV vaccines are currently licensed for use in Italy: RotaTeq (MSD Canada Inc., Rahway, NJ, United States) and Rotarix (GlaxoSmithKline, Brentford, United Kingdom). RotaTeq is a pentavalent human-bovine reassortant vaccine, which is administered in three doses beginning at 6 weeks of age, with a 4-week interval between doses, and ideally completed before the age of 20–22 weeks but no later than 32 weeks. Rotarix is a monovalent human vaccine, which follows a two-dose schedule, with the first dose administered at 6 weeks old and the second dose 4 weeks after the first (doses should be administrated by 16 weeks old and no later than 24 weeks old) [20].

Although the PNPV promotes uniform vaccination coverage throughout the country and underscores the importance of early childhood vaccinations, including for RV, it should be noted that Italy’s healthcare system is decentralised. Indeed, while being nationally regulated, healthcare in Italy is managed and implemented regionally. Regions have a considerable autonomy and can adapt the PNPV to carry it out according to local needs and resources. This leads to substantial disparities in vaccine coverage (VC), especially for recommended vaccinations.

Further to the PNPV recommending RV vaccination for all infants, several regions included this in their vaccination schedules [21]. However, to achieve nationwide protection against RV, addressing regional disparities and ensuring equitable access to vaccination are critical. Hence, this study aims to investigate vaccination policies and programmes in Italy, as well as the evolution and status of RV vaccination coverage across the country, focusing on both national and regional trends, and differences in vaccination schedules between regions.

## Methods

### Literature review of current policies

The review of current policies was conducted in accordance with the Interim Guidance from the Cochrane Rapid Reviews Methods Group [22]. Detailed methods are included in the registered and published protocol on Open Science Framework [23].

A systematic PubMed and Google search up to 2024 and without any other time restrictions was conducted to identify relevant documents published in either English or Italian that focused on free and universal RV vaccination policies or programmes in Italy at national level and in different Italian regions. All phases of study selection were carried out independently by two researchers, with disagreements resolved through discussion. From the included documents, we extracted information regarding the area (whole nation or specific region) in question, the year of the policy introduction or modification, key features of the policy, and, where available, strategies aimed at increasing vaccination uptake.

### Data collection and analysis

Data on vaccination coverage at both the national and regional levels in Italy were collected from the Italian Ministry of Health website [24] as they were in May 2025. A map of Italy with the Italian regions is available in the Supplementary Figure S1.

The percentage of 24-month-old children vaccinated is reported for each region. Available data refer only to children who completed the full rotavirus vaccine schedule within the recommended timeframe. We included data from 2016 (first data available) to 2023 (last data available). Data for Lombardia region were only available from 2019. Detailed demographic information on the Italian population and on children under 2 years old in each region is available in Supplementary Tables S2 and S3.

Descriptive analyses of VC over time were conducted in each region and at the national level, whereby we evaluated the percentage changes between the years considered and reported temporal trends. At regional level, we considered the rate change in coverage, from the first available measurement and the last, and the variation in terms of increase or decrease occurring during the last 3 years (2021, 2022, and 2023). 

To analyse the trend of VC both at national and regional level, a linear regression equation was applied and the coefficient of regression identified. We considered the vaccination coverage as the dependent variable, while the years represent the independent variable. Results were considered significant when p < 0.05. Statistical analyses were performed using Stata software, version 16 (StataCorp LP, College Station, TX).

## Results

### National and regional vaccination policies

**Figure 1:**
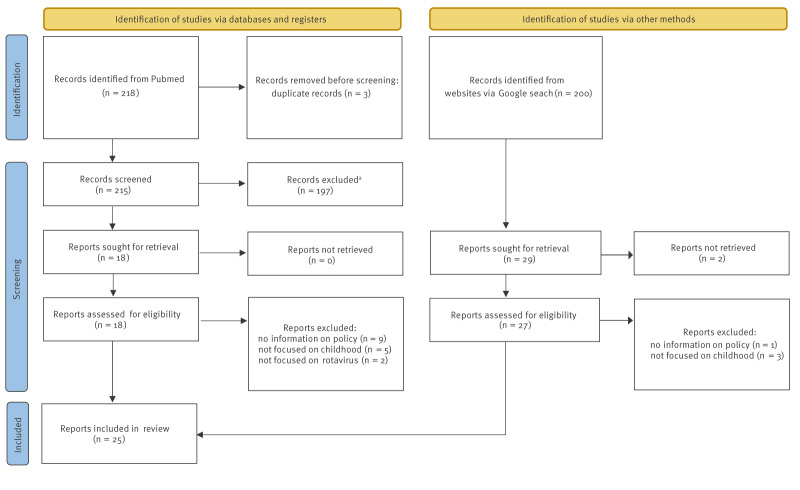
Preferred Reporting Items for Systematic reviews and Meta-Analyses (PRISMA) flow diagram to retrieve reports on national or regional policies related to rotavirus vaccination, Italy

^a^ In total, 197 records were excluded from the study, because despite having the relevant terms used for their search, they did not focus on national or regional policies related to rotavirus vaccination in Italy.

Among 418 records from scientific and grey literature, we included 25 documents regarding national or regional policies related to RV vaccination in Italy, as shown in the Preferred Reporting Items for Systematic reviews and Meta-Analyses (PRISMA) flow diagram (Figure 1). Furthermore, Table 1 lists the 25 unique sources; some are cited more than once, as certain documents provided information for multiple regions, while in other cases, multiple sources were identified for a single region.

**Table 1:** Rotavirus vaccination policies at national and regional levels, Italy, 2013–2024 (n=25 policies)

Region	Year	Key contents	References (URL)
**Northern regions**			
Piemonte	2017	▪RV vaccine offered free of charge to all newborns starting from the 6^th^ week of age	▪https://vaccinarsinpiemonte.org/scienza-conoscenza/vaccini-disponibili/vaccino-anti-rotavirus
Valle d'Aosta	2018	▪RV vaccine offered free of charge to all newborns starting from the 2018 cohort	▪https://www.regione.vda.it/sanita/prevenzione/igiene_sanita_pubblica/vaccinazioni/default_i.asp
Lombardia	2017	▪Administration of RV vaccine in combination with hexavalent and pneumococcal vaccines starting from the 3^rd^ month of life ▪RV vaccine offered free of charge to all newborns starting from the 2018 cohort ▪Guaranteeing requests for newborns from 2017, where consistent with the possibility of administration according to the technical data sheet	▪https://www.regione.lombardia.it/wps/wcm/connect/e5f8fbe7-d82d-46c0-912e-4ce3220446c3/DGR.7629_28.12.2017_Piano+Regionale+Prevenzione+Vaccinale+17_19.pdf?MOD=AJPERES&CACHEID=ROOTWORKSPACE-e5f8fbe7-d82d-46c0-912e-4ce3220446c3-n1sTFTb
AP Bolzano-Bozan	2017	▪RV vaccine offered free of charge based on the specific guidelines of the national vaccination schedule for each birth cohort	▪https://assets-eu-01.kc-usercontent.com/81954d07-82f8-0181-f3b0-51ccf4d64a1b/34772c9f-411a-4f79-b391-c672301102a8/Decreto_vaccini_n._73_2017%281%29.pdf
AP Trento	2017	▪RV vaccine offered free of charge based on the specific guidelines of the national vaccination schedule for each birth cohort	▪https://assets-eu-01.kc-usercontent.com/81954d07-82f8-0181-f3b0-51ccf4d64a1b/34772c9f-411a-4f79-b391-c672301102a8/Decreto_vaccini_n._73_2017%281%29.pdf
Veneto	2015	▪RV vaccine offered free of charge to all newborns starting from the 6^th^ week of age	▪https://www.vaccinarsinveneto.org/scienza-conoscenza/vaccini-disponibili/vaccino-anti-rotavirus
Friuli Venezia Giulia	2018	▪RV vaccine offered free of charge to all newborns since 2017 ▪Activation, starting in 2018, of reminders sent to parents by phone call or letter	▪https://www.ilfriuli.it/salute-e-benessere/in-fvg-arriva-la-vaccinazione-contro-il-rotavirus/
Liguria	2018	▪RV vaccine offered free of charge to all newborns starting from the 2^nd^ month of age (before 2018 there was a cost share)	▪https://www.regione.liguria.it/homepage-salute/cosa-cerchi/vaccinazioni.html ▪[26]
Emilia Romagna	2017	▪RV vaccine offered free of charge to all newborns starting from the 2^nd^ month of age	▪https://www.informafamiglie.it/salute-bambini/vaccinazioni
**Centre regions**			
Toscana	2018	▪RV vaccine offered free of charge to all newborns starting from the 6^th^ week of age ▪Administration in combination with hexavalent and meningococcal vaccine	▪https://www301.regione.toscana.it/bancadati/atti/Contenuto.xml?id=5426974&nomeFile=Delibera_n.777_del_01-07-2024-Allegato-B
Umbria	2018	▪RV vaccine offered free of charge based on the specific guidelines of the national vaccination schedule for each birth cohort	▪https://www.uslumbria1.it/servizio/vaccinazioni-bambini/
Marche	2017	▪RV vaccine offered free of charge to all newborns starting from the 2017 cohort	▪https://www.regione.marche.it/In-Primo-Piano/ComunicatiStampa?id=26357
Lazio	2018	▪RV vaccine offered free of charge to all newborns starting from the 6^th^ week of age	▪https://www.vaccinarsinlazio.org/scienza-conoscenza/vaccini-disponibili/vaccino-anti-rotavirus
**Southern regions**			
Abruzzo	2017	▪RV vaccine offered free of charge to all newborns starting from the 2017 cohort	▪https://lnx.asl2abruzzo.it/formazione/attachments/article/415/Vaccini%20pacchetto.pdf
Molise	2018	▪RV vaccine offered free of charge to all newborns starting from the 6^th^ week of age ▪Administration in combination with other age-matched vaccines	▪https://www.google.com/url?sa=t&source=web&rct=j&opi=89978449&url=https://www3.regione.molise.it/flex/cm/pages/ServeAttachment.php/L/IT/D/4%25252Fd%25252F5%25252FD.f9857da5a2b5fb8d884f/P/BLOB%253AID%253D15564/E/pdf&ved=2ahUKEwiQkby8842KAxUrzwIHHZwRDF8QFnoECA4QAQ&usg=AOvVaw1Ob2H61pfrrxbimQ7pGcdP
Campania	2018	▪RV vaccine offered free of charge based on the specific guidelines of the national vaccination schedule for each birth cohort	▪https://www.vaccinarsincampania.org/scienza-conoscenza/vaccini-disponibili/fogli-illustrativi-schede-tecniche-vaccini
Puglia	2015	▪RV vaccine offered free of charge to all newborns starting from the 6^th^ week of age	▪https://www.vaccinarsinpuglia.org/scienza-conoscenza/vaccini-disponibili/vaccino-anti-rotavirus
Basilicata	2018	▪RV vaccine offered free of charge to all newborns starting from the 6^th^ week of age	▪https://www.asmbasilicata.it/upload/asm_matera/gestionedocumentale/OPUSCOLO2EdizioneProntuariodelVaccinatore2018_784_3897.pdf
Calabria	2015	▪RV vaccine offered free of charge based on the specific guidelines of the national vaccination schedule for each birth cohort ▪Administration in combination with other age-matched vaccines	▪https://www.regione.calabria.it/wp-content/uploads/2022/04/all.-dca-32-del-7.4.2022-_note-esplicative-calendario-vaccinale.pdf
Sicilia	2013	▪RV vaccine offered free of charge to all newborns starting from the 6^th^ week of age	▪https://www.vaccinarsi.org/notizie/2014/08/28/antirotavirus-in-sicilia#:~:text=In%20Italia%20solo%20la%20Regione,il%20vaccino%20non%20viene%20offerto. ▪[27]
Sardegna	2016	▪RV vaccine offered free of charge based on the specific guidelines of the national vaccination schedule for each birth cohort	▪https://www.regione.sardegna.it/documenti/1_386_20171212135626.pdf
**Italy**			
Italy	2017	▪The National Vaccination Plan (PNPV) 2017–2019 recommended rotavirus vaccination for infants in their first year of life and offers it free of charge^a^. ▪The National Vaccination Plan (PNPV) 2023–2025 confirms the recommendation of rotavirus vaccination for all infants and maintains its free availability.	▪https://www.salute.gov.it/imgs/C_17_pubblicazioni_2571_allegato.pdf ▪https://www.epicentro.iss.it/piano_prevenzione/pnp-2020-25

AP: autonomous province; hexavalent vaccine: vaccine against diphtheria, *Haemophilus influenzae* type b infection, hepatitis B, pertussis, poliomyelitis and tetanus; RV: rotavirus.

^a^ With the COVID-19 pandemic, the PNPV 2017−19 was extended first to 2021 and then until the approval of the new PNPV 2023−25.

The RV vaccine was included into the PNPV for the years 2017–19 [25], in accordance with the legislative decree n° 73 of 2017 [18]. With the COVID-19 pandemic, the PNPV 2017–19 was extended first to 2021 and then until the approval of the new PNPV 2023–25. Following the 2017 PNPV introduction, offering the rotavirus vaccine free of charge became mandatory according to the vaccination schedule, and all regions were required to comply with this regulation with a specific regional law. This policy was confirmed by the new PNPV 2023–25.

Prior to the PNPV, the availability of the RV vaccine varied considerably among regions (Table 1). While some regions restricted its access to specific high-risk groups, such as premature infants or children attending daycare others employed a copayment system. The Piemonte region was the first to offer the vaccine with a copayment in 2010. A similar approach was then adopted by the Friuli-Venezia Giulia and Liguria regions as well as the Autonomous Province (AP) of Bolzano in 2013 [26]. Subsequently the Veneto and Puglia regions also followed this model in 2014. The first region to implement an active, free and universal RV vaccination initiative was Sicilia in 2013 [27], followed by Calabria and Puglia in 2015, and Sardegna in 2016. Between 2017 and 2018, all regions and APs progressively adopted a more uniform approach, making the RV vaccine universally and freely accessible to all eligible newborns.

### Vaccination coverage in Italy at national level

**Figure 2:**
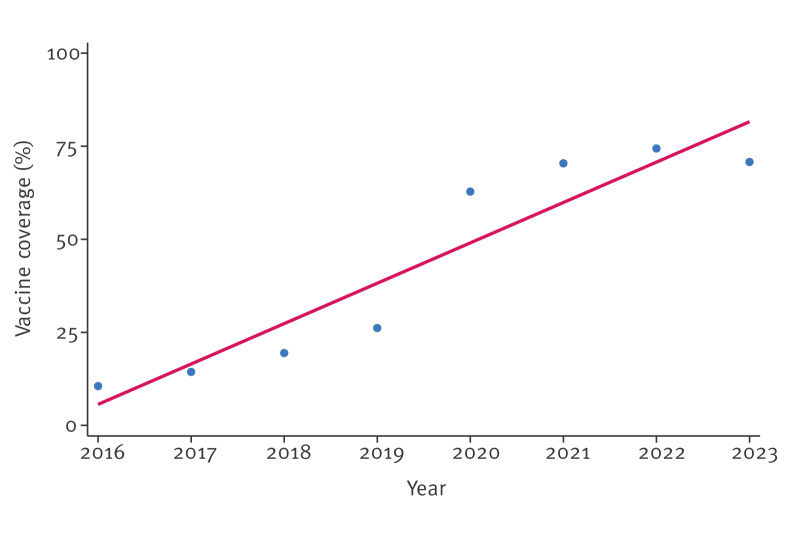
Linear regression for annual changes in vaccination coverage^a^ in Italy, 2016–2023

^a^ The vaccination coverage is the percentage of 24-month-old children who completed the full rotavirus vaccination schedule within the recommended timeframe.

Data on RV vaccination in Italy is available from 2016 to 2023 (Table 2), and a progressive increase in VC can be observed over the years, albeit with a reduction from 2022 to 2023. Overall, the linear regression showed a significant VC increase over the entire period (regression coefficient: 10.85; 95% confidence interval (CI): 7.66–14.03, p = 0.0005) (Figure 2).

**Table 2:** Rotavirus vaccination coverages^a^ in the country and by regions (n = 21), Italy, 2016–2023

Regions	Vaccine coverage (%)^a^ in year							
	2016	2017	2018	2019	2020	2021	2022	2023
Piemonte	6.39	8.50	11.47	18.68	75.81	78.40	80.29	81.06
Valle d’Aosta	0.00	0.21	0.94	1.13	23.71	40.23	59.71	70.18
Lombardia	n.d.	n.d.	n.d.	8.99	74.21	79.68	84.32	82.9
AP Bolzano	0.91	1.03	2.72	23.63	41.69	39.68	38.12	48.58
AP Trento	0.00	0.73	0.94	2.60	72.89	78.68	74.03	75.24
Veneto	2.53	6.26	11.23	25.99	80.95	85.10	84.96	84.91
Friuli Venezia Giulia	2.01	4.09	9.47	22.3	66.57	75.28	76.52	81.09
Liguria	16.76	20.07	19.67	39.68	58.08	68.19	69.27	71.31
Emilia-Romagna	3.76	6.22	9.69	26.34	73.45	76.16	78.38	68.02
Toscana	8.14	8.27	10.16	23.25	43.95	57.76	64.73	66.92
Umbria	0.00	0.00	0.25	1.04	27.62	51.88	62.01	65.57
Marche	0.53	1.51	2.69	8.77	58.99	67.38	71.45	68.09
Lazio	7.01	7.44	18.69	38.43	43.73	60.09	65.49	66.23
Abruzzo	0.52	1.02	1.00	13.44	44.46	58.72	62.01	65.2
Molise	0.00	0.09	0.14	1.20	73.97	78.04	83.02	83.94
Campania	0.33	0.76	1.56	4.00	39.89	59.71	65.56	51.33
Puglia	21.12	29.71	35.04	53.04	69.57	76.77	79.45	60.71
Basilicata	1.35	2.56	4.41	24.18	65.12	76.65	80.17	78.7
Calabria	14.64	41.52	75.55	72.43	79.91	77.18	83.49	79.59
Sicilia	45.06	50.92	53.77	54.29	59.83	59.49	64.40	62.39
Sardegna	13.55	21.50	29.52	35.93	79.19	75.20	77.96	79.16
**Italy**	**10.55**	**14.36**	**19.44**	**26.15**	**62.80**	**70.40**	**74.39**	**70.76**

AP: autonomous province; n.d.: no data.

^a^ The vaccination coverage is the percentage of 24-month-old children who completed the full rotavirus vaccination schedule within the recommended timeframe.

From 2016 to 2019, coverage rose from 10.55% in 2016 to 14.36% in 2017, 19.44% in 2018, and 26.15% in 2019. In 2020, a relevant rise in coverage was observed (+ 140%) compared with 2019, reaching 62.80% of the target population. This upward trend continued in the most recent years, with a coverage of 70.40% in 2021 and 74.39% in 2022. A reduction of − 4.88% was observed in 2023 (70.76%). Overall, an increase of + 570.71% was observed during the period considered.

### Vaccination coverage by regions

**Figure 3:**
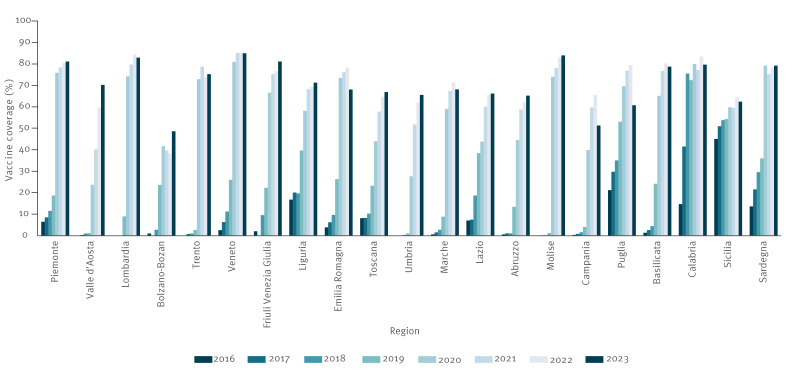
Annual rotavirus vaccination coverage by regions, Italy, 2016–2023 (n = 21 regions)

The regional situation was very varied, with also fluctuations over time (Table 1, Figure 3). Between 2016 and 2023, a statistically significant annual increase of VC occurred in all regions, except in Lombardia, where data were only available from 2019 and the trend, while seemingly positive, was not statistically significant. The statistical analysis is presented in the Supplementary Table S1 and the trends illustrated in Figure S2.

Data for Lombardia are available from 2019.

During the study period, Sicilia showed the least fluctuations in vaccination coverage (from 45.06% in 2016 to 62.39% in 2023). This region, however, started from higher coverage values than all the other regions. Of all regions in 2016–23, the highest coverage (85.10%) was reached in Veneto in 2021, although this was followed by a slight decrease in 2022 (84.96%) and in 2023 (84.91%). The highest increases in coverage were observed in Veneto (from 2.53% in 2016 to 84.91% in 2023) and Molise (from 0% in 2016 to 83.94% in 2023).

**Figure 4:**
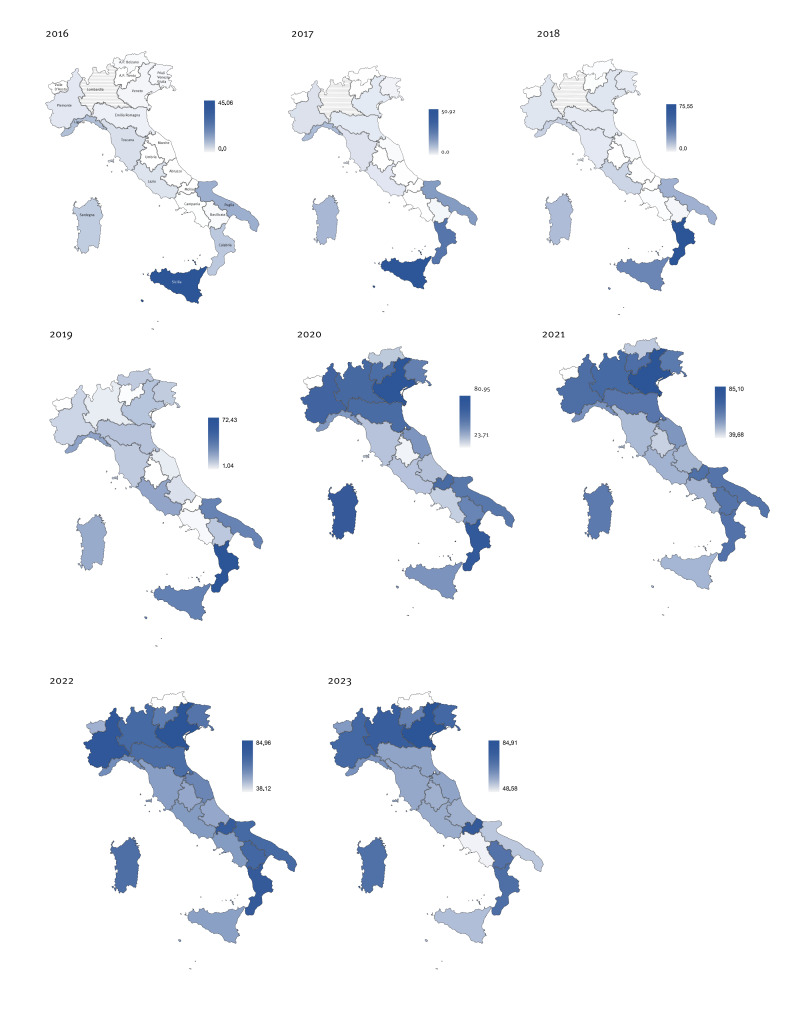
Maps of regional vaccination coverage across the country, by region, Italy, 2016–2023

In 2016, only five regions (Sicilia, Puglia, Liguria, Calabria and Sardegna) appeared to have a higher value than the national average (10.55%). At this time, coverage, varied from 0% to a maximum of 45.06% in Sicilia. While coverage generally increased both at national and regional level in 2017 and 2018, the regional distribution remained similar, with the same five regions showing VC values above the Italian average. In 2019, coverage ranged from a low of 1.04% in Umbria to a high of 72.43% in Calabria, with a third of the regions having coverage above the national value (26.15%). In 2020, a notable increase in coverage was observed (Figure 4), with regional coverage varying between 23.71 (Valle d’Aosta) and 80.95 (Veneto). Two-thirds of regions reported coverage rates above 50%, and among the remaining, VC in Abruzzo, AP Bolzano, Toscana, Lazio, and Campania was between 30% and 50%, whereas Valle d’Aosta and Umbria had a coverage below 30%. 

A.P.: autonomous province.

The number of regions with coverage over 50% further increased in 2021, during the COVID-19 pandemic, when only two regions with a VC below 50% remained (Valle d’Aosta and AP Bolzano). As of 2022, all regions except AP Bolzano had a VC above 50%, with six regions achieving a VC above 80%. In 2023, only AP Bolzano had coverage below 50% (48.58%), even though a notable increase was observed in this province compared with 2022 (+ 27.43%). In 2023, a decrease in coverage was observed compared to 2022 in nine regions, but mainly in Campania (− 21.71%) and Puglia (− 23.59%). Five regions had VC above 80%.

At the macro area level, while between 2016 and 2023 VC values in the north and south regions were distributed either above or below the national value, all central regions, except Lazio in 2019, consistently reported values below the national value (Supplementary Figure S3).

## Discussion

This study sheds light on the evolving landscape of RV vaccination in Italy, revealing an important progress in national coverage over recent years but persistent regional disparities. Nationally, RV vaccination coverage increased substantially from 10.55% in 2016 to 70.76% in 2023, driven by the inclusion of the vaccine in the PNPV [28]. The PNPV 2017–2019 recommended RV vaccination for all infants, aiming to standardise immunisation across the country and ensure equitable protection. However, the decentralised healthcare system, which grants each region autonomy in implementing vaccination policies, has led to marked differences in vaccine accessibility and uptake [25].

An analysis of the regional data shows substantial variability. Veneto and Molise exhibited the most notable VC increases, with coverage rising from 2016 to 2023 from 2.53% and 0%, respectively, to over 83%, underscoring the impact of proactive policies and effective implementation strategies [29]. Conversely, Bolzano with a maximum rate of 48.58% in 2023 was the only region to not reach at least 50% in coverage rate in any year. These data reflect systemic and cultural challenges, including a longstanding mistrust in vaccines [30]. In Sicilia, an early adopter of universal vaccination policies, the annual coverage rates kept above the respective national values until 2017, thereafter dropping below these. While the region’s early commitment to RV vaccination ensured a strong baseline, the pace of improvement was slower compared with other regions like Veneto.

Indeed, differences in regional policies played a central role in shaping VC trends. Before the inclusion of RV vaccination in the PNPV, early adopters like Sicilia, Calabria, and Puglia had already implemented universal and free of charge programmes between 2013 and 2015 [27], ensuring higher initial coverage rates. By contrast, Veneto initially employed a copayment model for the vaccination target population and transitioned to universal access for free only after 2017 [29]. Despite this delayed adoption, Veneto has now reached the highest national coverage rate, highlighting the importance of robust implementation strategies and public engagement. In central Italy, regions such as Umbria and Marche lagged behind, consistently reporting coverage below the national values. This underperformance likely reflects slower adoptions of universal policies and, potentially, less effective outreach efforts to promote awareness among the public [31].

Low vaccine acceptance also emerges as a critical factor influencing coverage, particularly in regions like AP Bolzano, where unfavourable attitudes in paediatric vaccines have historically been high [32,33]. The optional status of RV vaccination, compared with mandatory vaccines, may further undermine its perceived importance, despite its proven effectiveness in reducing hospitalisations and severe disease [34]. Moreover, the strict vaccination schedule, which requires doses to be administered within a narrow timeframe to minimise risks like intussusception, poses logistical challenges. Infants who do not complete the full course on time are excluded from coverage statistics, potentially underestimating the actual vaccination efforts [35].

The COVID-19 pandemic introduced additional challenges but also revealed opportunities. Although routine vaccination programmes faced disruptions worldwide, Italy’s RV vaccination rates continued to rise during 2021 and 2022, while a decrease in nine regions was observed in 2023. This resilience may reflect increased public awareness of infectious diseases during the pandemic, which positively influenced vaccine uptake. However, the pandemic’s long-term impact on vaccine trust and hesitancy, particularly for non-mandatory vaccines like RV, remains uncertain and warrants further monitoring [32].

Finally, this study underscores the importance of education and outreach in addressing disparities [36]. Vaccination against RV is a safe and effective intervention that reduces hospitalisations, mortality, and the economic impact of disease. Targeted efforts to inform healthcare providers and the population about these benefits, while addressing vaccine hesitancy and logistical challenges, are essential [37]. A harmonised national approach, supported by consistent regional efforts, is crucial to bridge gaps and achieve equitable protection against RV across Italy [38].

Our study has some limitations. First, the search for RV vaccination policies in Italy may not have been exhaustive, although the methodology used ensures a systematic search of policies, including both grey and scientific literature. Regarding the vaccination data obtained, it was also not possible to conduct inferential or more detailed descriptive analyses of trends as the period considered is limited. Vaccination data included only children who completed the full rotavirus vaccine schedule on time, which may slightly underestimate overall uptake. However, the data are taken from official sources and our study analyses trends in RV vaccination coverage for the first time from the first available years. It does provide evidence-based coverage trends and assess regional differences and progress over the years. Finally, given the limited period of the analysis, the linear regression results warrant cautious interpretation. Nevertheless, the analysis elucidates trends at both national and regional levels, providing a broad characterisation of coverage patterns.

## Conclusions

This study highlights relevant progress in RV vaccination coverage in Italy while emphasising persistent regional disparities that reflect differences in policy adoption, healthcare organisation, and public attitudes rather than simple geographical patterns. The ultimate goal should be to reach the highest possible coverage across all regions. Nevertheless, a cohesive national public health strategy must be pursued in order to achieve equitable access to vaccination and reach uniformly high coverage over the country. This would include training paediatricians and general practitioners to reduce low vaccine acceptance, enhance their knowledge, and encourage them to recommend the RV vaccine during routine visits. Importantly, a coordinated strategy would also actively involve both the general population and parents to raise their awareness of vaccination, explain its benefits, and address unfavourable attitudes toward vaccination. Moreover, integrating RV vaccination during other scheduled immunisation appointments, as well as organising vaccination days in schools and daycares could help facilitate scheduling for parents and strengthen their commitment.

Achieving this target will require harmonised policies, enhanced education efforts, and targeted interventions to address logistical and societal challenges. These measures are essential to reduce the RV infections and strengthen the equity and effectiveness of Italy’s public health system.

## Data Availability

The data are obtained from the Italian Ministry of Health and are public and free.

## Supplementary Data

833000 SupplementaryMaterial
