# Supplementary material for: A review of childhood rotavirus vaccination policies and a presentation of vaccine coverage trends at national and regional level, Italy, 2016 to 2023
Source: Euro Surveill. 2025 Oct 2;30(39):2500026. doi: 10.2807/1560-7917.ES.2025.30.39.2500026 (PMC12495378; doi:10.2807/1560-7917.ES.2025.30.39.2500026)

This supplementary material is hosted by Eurosurveillance as supporting information alongside the article "A review of childhood rotavirus vaccination policies and a presentation of vaccine coverage trends at national and regional level, Italy, 2016 to 2023", on behalf of the authors, who remain responsible for the accuracy and appropriateness of the content. The same standards for ethics, copyright, attributions and permissions as for the article apply. Supplements are not edited by Eurosurveillance and the journal is not responsible for the maintenance of any links or email addresses provided therein.

Supplementary Figure S1. Italian regions

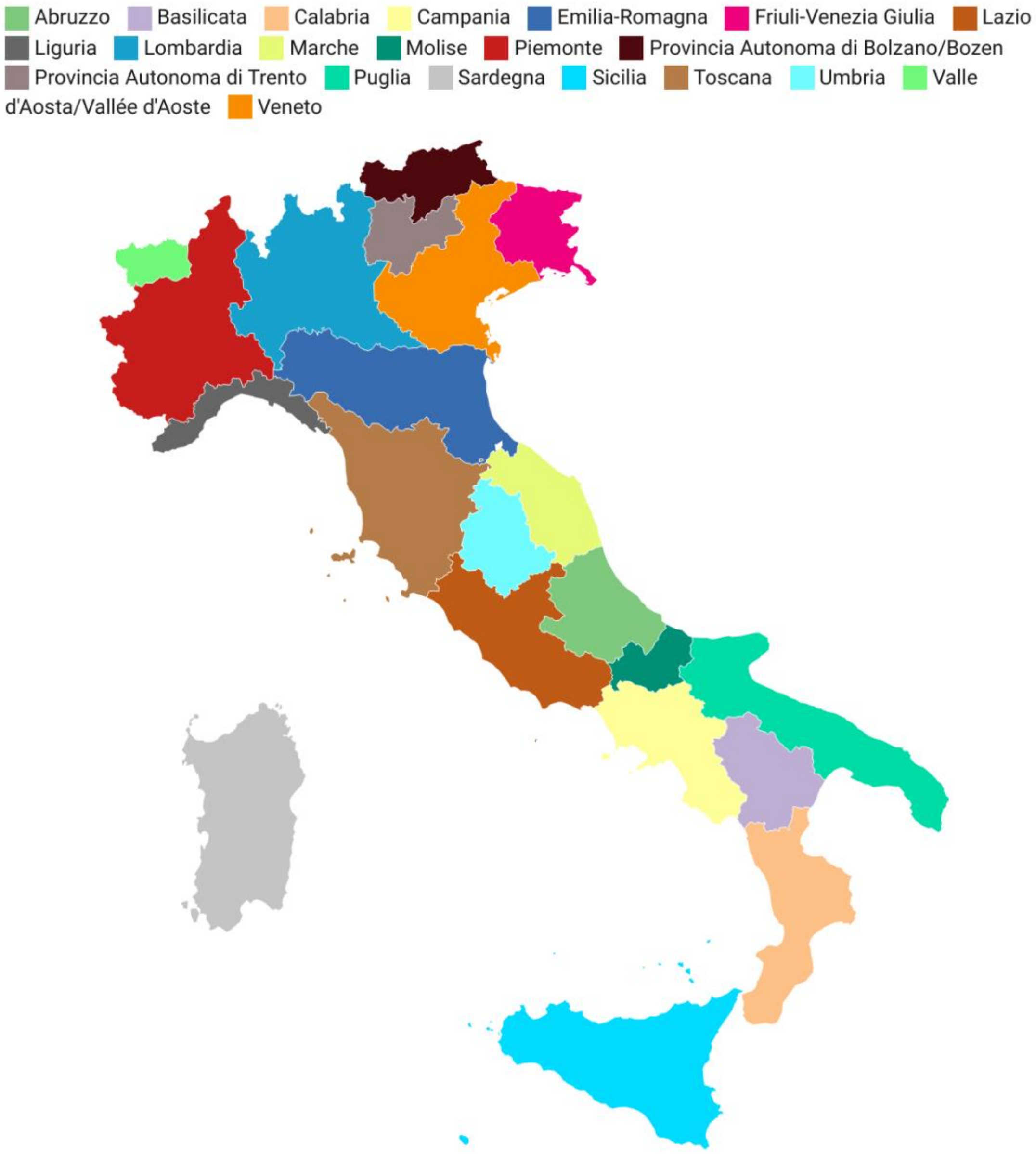

Note for the figure:

Macroregions:

North: Piemonte, Valle d'Aosta, Lombardia, Liguria, A.P. Bolzano, A.P. Trento, Veneto, Friuli-Venezia Giulia, Emilia-Romagna

Centre: Toscana, Umbria, Marche, Lazio

South and Island: Abruzzo, Molise, Campania, Puglia, Basilicata, Calabria, Sicilia, Sardegna

Supplementary Table S1. Linear Regression Results for Annual Changes in Vaccination Coverage by Region and in Italy (2016–2023)

Region	CR	Lower 95%CI	Upper 95%CI	p_value
Piemonte	13.57	8.75	18.38	0.0015
Valle d'Aosta	11.06	7.73	14.39	0.0006
Lombardia	15.79	1.34	30.25	0.076
Liguria	9.43	7.13	11.72	0.0002
A.P. Bolzano	7.72	5.33	10.1	0.0007
A.P. Trento	14.25	8.08	20.41	0.004
Veneto	14.84	9.93	19.76	0.001
Friuli Venezia Giulia	13.78	9.99	17.56	0.0004
Emilia-Romagna	12.59	7.75	17.42	0.0022
Toscana	10.21	8.01	12.4	0.0001
Umbria	11.32	7.83	14.8	0.0007
Marche	12.7	8.23	17.18	0.0014
Lazio	9.93	8.2	11.66	0.0001
Abruzzo	11.45	8.4	14.5	0.0003
Molise	15.58	9.44	21.72	0.0025
Campania	10.61	6.41	14.81	0.0026
Puglia	7.95	4.4	11.5	0.0046
Basilicata	14.13	10.06	18.2	0.0005
Calabria	8.06	3.36	12.76	0.0152
Sicilia	2.52	1.9	3.13	0.0002
Sardegna	10.97	7.48	14.47	0.0008
**Italy**	**10.85**	**7.66**	**14.03**	**0.0005**

CR= coefficient of regression

CI = Confident interval

Supplementary Table S2. Resident population of Italy and by region as of December 31 of each year (2016-2023)

Region	2016	2017	2018	2019	2020	2021	2022	2023
Piemonte	4,384,226	4,370,348	4,349,911	4,328,565	4,311,217	4,274,945	4,251,351	4,251,623
Valle d'Aosta	127,030	126,677	126,213	125,653	125,034	124,089	123,130	122,877
Lombardia	9,958,447	9,970,419	9,986,962	10,010,833	10,027,602	9,981,554	9,976,509	10,012,054
Liguria	1,557,742	1,551,379	1,541,541	1,532,980	1,524,826	1,518,495	1,507,636	1,509,140
Trentino-Alto Adige/Südtirol [a]	1,059,765	1,063,734	1,068,738	1,074,034	1,078,069	1,086,095	1,086,143	1,082,702
Veneto	4,890,648	4,883,373	4,880,936	4,884,590	4,879,133	4,847,745	4,849,553	4,852,216
Friuli Venezia Giulia	1,216,208	1,212,809	1,211,155	1,210,414	1,206,216	1,194,647	1,194,248	1,194,616
Emilia-Romagna	4,435,480	4,439,768	4,445,920	4,459,453	4,464,119	4,438,937	4,437,578	4,451,938
Toscana	3,726,422	3,721,391	3,712,048	3,701,343	3,692,555	3,692,865	3,661,981	3,660,530
Umbria	884,092	880,992	876,477	873,744	870,165	865,452	856,407	853,068
Marche	1,538,442	1,532,460	1,526,331	1,520,321	1,512,672	1,498,236	1,484,298	1,482,746
Lazio	5,761,508	5,774,092	5,774,606	5,773,076	5,755,700	5,730,399	5,720,536	5,714,745
Abruzzo	1,319,294	1,313,930	1,306,059	1,300,645	1,293,941	1,281,012	1,272,627	1,269,571
Molise	310,026	308,400	306,564	303,790	300,516	294,294	290,636	289,224
Campania	5,790,783	5,776,654	5,762,889	5,740,291	5,712,143	5,624,260	5,609,536	5,593,906
Puglia	4,043,735	4,024,067	4,000,966	3,975,528	3,953,305	3,933,777	3,907,683	3,890,661
Basilicata	569,887	566,405	562,968	558,587	553,254	545,130	537,577	533,233
Calabria	1,943,085	1,935,097	1,924,257	1,912,021	1,894,110	1,860,601	1,846,610	1,838,568
Sicilia	5,004,400	4,977,900	4,942,188	4,908,548	4,875,290	4,833,705	4,814,016	4,797,359
Sardegna	1,642,492	1,636,839	1,631,040	1,622,257	1,611,621	1,590,044	1,578,146	1,570,453
Italy	60,163,712	60,066,734	59,937,769	59,816,673	59,641,488	59,236,213	58,997,201	58,971,230

[a]= A.P- Bolzano and A.P. Trento together represent the Trentino Alto Adige region,

Supplementary Table S3. Children under 2 years old in Italy and by region as of December 31 of each year (2016-2023)

Region	2016	2017	2018	2019	2020	2021	2022	2023
Piemonte	102,647	98,844	95,368	91,226	87,614	84,706	82,483	80,659
Valle d'Aosta	3,127	3,003	2,844	2,733	2,616	2,507	2,350	2,316
Lombardia	258,177	251,428	244,347	236,096	228,202	220,126	212,954	208,118
Liguria	31,746	30,708	29,516	28,281	27,277	26,857	26,427	26,258
Trentino-Alto Adige/Südtirol [a]	30,999	30,718	30,233	29,840	29,228	28,560	28,223	27,687
Veneto	120,944	117,035	113,304	109,796	105,598	102,329	99,675	98,147
Friuli Venezia Giulia	27,053	26,161	25,202	24,522	23,481	22,924	22,468	22,287
Emilia-Romagna	110,753	107,492	103,885	100,897	97,432	94,317	91,637	90,556
Toscana	85,900	83,281	80,303	77,362	74,045	71,160	68,883	67,350
Umbria	20,663	19,707	18,791	18,034	17,179	8,108	16,194	15,566
Marche	36,708	35,454	33,850	32,114	30,428	29,447	28,554	27,842
Lazio	149,802	145,068	139,054	130,624	124,798	121,710	116,514	113,535
Abruzzo	31,056	30,408	29,387	28,062	26,618	25,765	25,208	24,803
Molise	6,549	6,431	6,281	5,984	5,830	5,474	5,274	5,076
Campania	153,967	151,280	149,648	146,061	142,494	139,378	135,096	132,915
Puglia	97,271	94,574	91,522	88,474	85,189	82,855	80,557	79,404
Basilicata	12,195	12,070	11,966	11,522	11,167	10,804	10,469	10,043
Calabria	48,826	48,223	47,331	45,904	44,299	43,383	41,534	40,648
Sicilia	130,481	127,465	123,994	120,489	117,644	116,343	113,541	111,916
Sardegna	33,937	32,681	31,462	29,545	27,934	26,447	25,402	24,293
Italy	1,492,801	1,452,031	1,408,288	1,357,566	1,309,073	1,271,796	1,233,443	1,209,419

[a]= A.P- Bolzano and A.P. Trento together represent the Trentino Alto Adige region,

Supplementary figure S2. Linear regression results for annual changes in vaccination coverage by region (2016–2023)

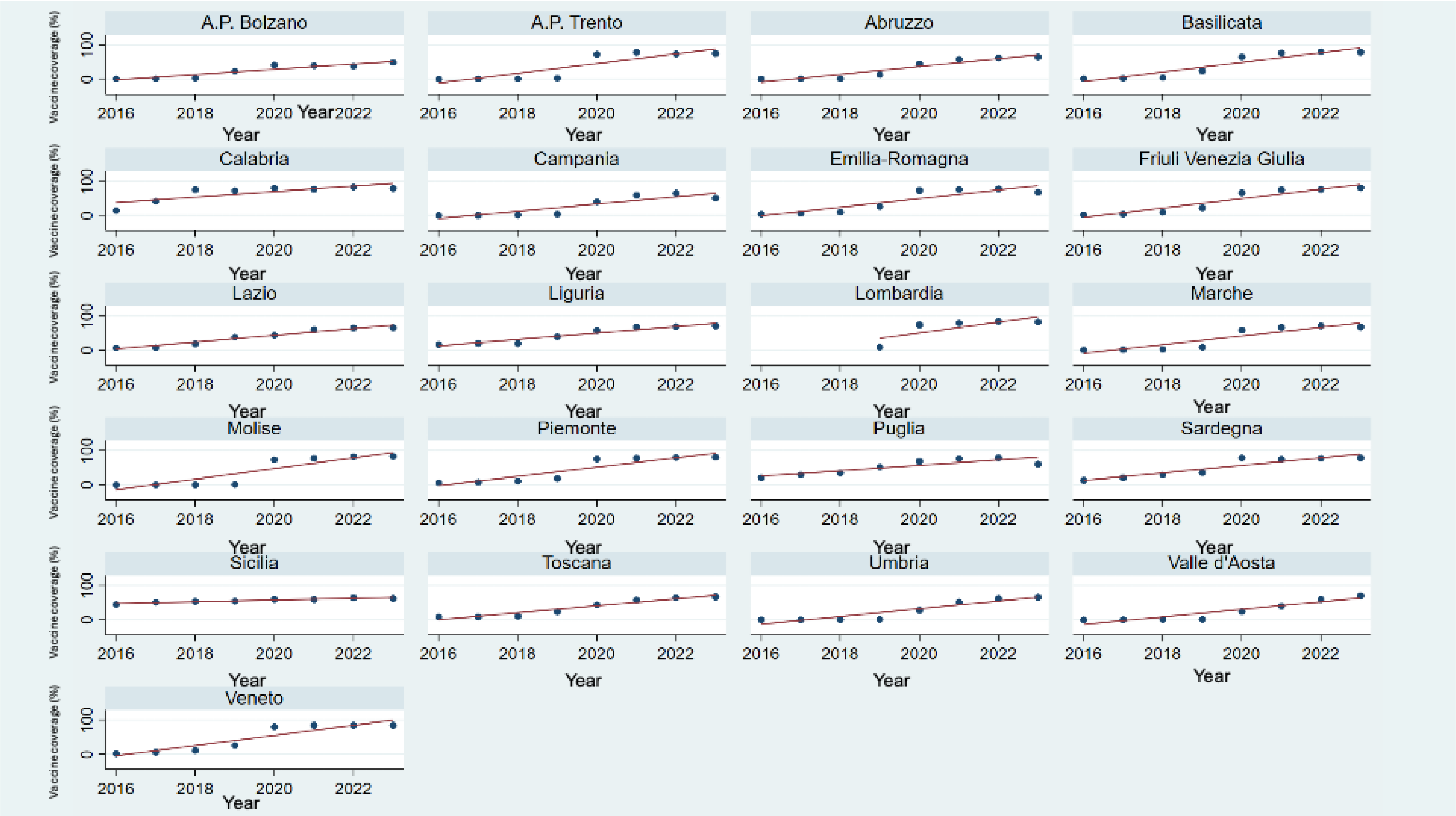

Supplementary figure S3. Rotavirus vaccination coverages by macro areas, in Italy, 2016-2023.

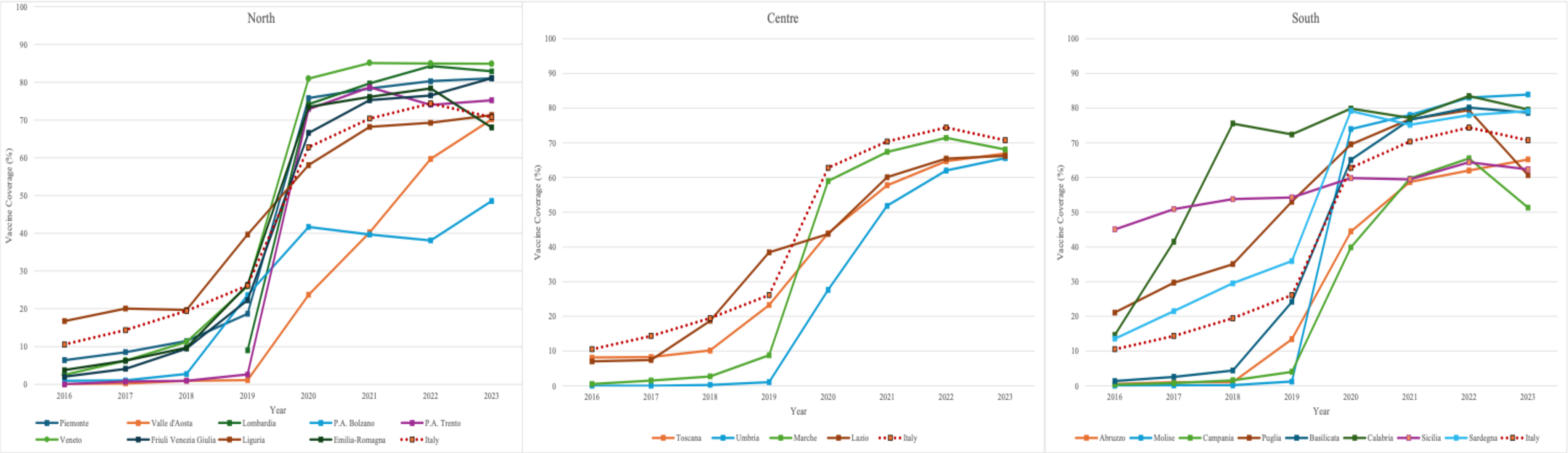

Supplement: SupplementaryMaterial [file 24-00026_SupplementaryMaterial.pdf]
